# 肺癌干性样细胞与耐药

**DOI:** 10.3779/j.issn.1009-3419.2022.102.02

**Published:** 2022-02-20

**Authors:** 振华 潘, 红雨 刘, 军 陈

**Affiliations:** 1 300052 天津，天津医科大学总医院，天津市肺癌研究 所，天津市肺癌转移与肿瘤微环境重点实验室 Tianjin Key Laboratory of Lung Cancer Metastasis and Tumor Microenvironment, Tianjin Lung Cancer Institute, Tianjin Medical University General Hospital, Tianjin 300052, China; 2 300052 天津，天津医科大学总医院肺部肿瘤外科 Department of Lung Cancer Surgery, Tianjin Medical University General Hospital, Tianjin 300052, China

**Keywords:** 肺肿瘤, 干性样细胞, 耐药, 靶向, Lung neoplasms, Stem-like cells, Drug resistance, Therapy

## Abstract

肺癌是全球致死率最高的肿瘤，耐药和复发是肺癌致死率居高不下的关键原因之一。大量研究证据表明，在肺癌组织和细胞中存在一小群具有干性样特征的细胞，这群细胞被证实能够自我更新、多向分化和无限增殖，在体内可以高效成瘤，能在放疗和化疗中存活，最终引起肺癌的耐药和复发。本文着重针对肺癌干性样细胞的来源、分子生物学特征和鉴定方法以及广谱耐药机制进行综述，旨在为进一步研究其临床诊断和靶向策略提供依据。

## Abstract (en)

Lung cancer remains the leading cause of cancer-related death world-wide. Therapy resistance and relapse are considered major reasons contributing to the poor survival rates of lung cancer. Accumulated evidences have demonstrated that a small subpopulation of stem-like cells existed within lung cancer tissues and cell lines, possessing the abilities of self-renewal, multipotent differentiation and unlimited proliferation. These lung cancer stem-like cells (LCSCs) can generate tumors with high effeciency *in vivo*, survive cytotoxic therapies, and eventually lead to therapy resistance and recurrence. In this review, we would like to present recent knowledges on LCSCs, including the origins where they come from, the molecular features to identify them, and key mechanisms for them to survive and develop resistance, in order to provide a better view for targeting them in future clinic.

据世界卫生组织发布的2020年GLOBOCAN数据显示，肺癌是全球发病率第二、致死率第一的肿瘤，其中非小细胞肺癌（non-small cell lung cancer, NSCLC）占85%，小细胞肺癌（small cell lung cancer, SCLC）占15%。按照组织类型，NSCLC又分为肺腺癌、肺鳞癌和大细胞肺癌。对于中晚期的肺癌患者，单独或者联合化疗是目前临床的主要策略，尽管靶向治疗和免疫治疗大大改善了部分患者的生存状态，但无论对于传统化疗还是新型疗法，耐药和复发仍然使肺癌临床面临困境^[1-3]^。

肿瘤干细胞（cancer stem cells, CSCs）最早在急性髓系白血病中得到鉴定，随后研究者们陆续在多种实体瘤中证实了CSCs的存在，并提出CSCs与肿瘤的发生、转移、耐药和复发密切相关^[4]^。2007年Maria的课题组^[5]^利用干细胞对Hoechst 33342染料的高外排能力从人肺癌细胞株和肺癌组织中均分离出了具有干性样特征的细胞亚群，证实了这群细胞能够自我更新和多向分化，在体内可以高效成瘤。2008年Levina的课题组^[6]^发现在多种化疗药物作用下存活下来的肺癌细胞高表达干性标志蛋白，同时这群细胞具有自我更新和体外成球等干性特征。近年来，更多的证据表明在肺癌组织和细胞中存在一小群干性样肿瘤细胞，它们可以自我更新、多向分化，具有很强的成瘤能力，能够在放疗、化疗、靶向治疗和免疫治疗中存活，是肺癌耐药与复发的主要原因^[7]^。

本文主要针对肺癌干性样细胞（lung cancer stem-like cells, LCSCs）及其耐药机制的研究进展进行综述，旨在为临床靶向和逆转耐药提供思路。

## 1 肺癌干性样细胞

### 1.1 来源

目前研究者们认为LCSCs的来源主要包括：正常成体干细胞或者前体细胞发生恶性转化，以及已分化的肿瘤细胞通过去分化获得干性。近来有团队提出，在肿瘤组织中可能不仅仅存在单一的CSC亚群，而可能有多个亚群，它们可能是单来源，也可能是多来源，其恶性转化途径可能既存在重合，也存在各自的特异性，这对于干性样肿瘤细胞的鉴定与靶向提出了更加严峻的挑战^[7]^。

#### 1.1.1 成体干细胞来源的LCSCs

研究最多的肺部成体干细胞包括基底细胞、Ⅱ型肺泡表皮（alveolar epithelial type Ⅱ, AT2）细胞和club细胞，它们除维系肺部组织平衡发育，在损伤修复中也发挥重要功能。研究^[7]^表明，细胞自身的DNA损伤修复应答、基因突变和炎性环境的诱导等多种因素可能引起这些成体干细胞在分裂和增殖过程中累积突变而发生恶性转化。

Weeden团队^[8]^发现基底细胞在吸烟等引起的肺损伤时更倾向于启动非同源末端连接（non-homogenous end joining, NHEJ）途径进行修复，从而增加了基因组的不稳定性和突变几率，基因表达分析进一步论证了肺鳞癌与基底细胞之间具有高度同源性，这为肺鳞癌的基底细胞来源提供了有力的证据。Jeong团队^[9]^通过*Trp53*和*Keap1*双突变，诱导小鼠气道基底干细胞发生了向肺鳞癌的恶性转化，证实了肺鳞癌可由基底细胞恶性突变形成，并成功建立了小鼠基底细胞发展为肺鳞癌的模型。

通过小鼠模型，研究者们追踪到在出生后，肺部损伤可以诱导AT2细胞分化产生新的AT1细胞，同时，体外添加表皮生长因子（epidermal growth factor, EGF）等可以诱导AT2细胞的自我更新。在*Kras^G12D^*突变型小鼠体内的AT2细胞可以被有效地激发出自我更新能力，并进一步发展为多灶位肺腺癌^[10]^。在最新的研究中，Chen的团队^[11]^通过类器官模型证实表皮生长因子受体（epidermal growth factor receptor, *EGFR*）T790M/L858R突变的AT2细胞和支气管肺泡来源的干性细胞能够转化为肺癌细胞，转化后的癌细胞保留了它们各自的表观遗传学特征，并对不同的药物表现出不同的敏感性。

当肺部受损严重，或者采用基因编辑的方式去除掉club细胞时，通常保持休眠状态的神经内分泌（neuroendocrine, NE）细胞就会发生增殖并对周围表皮细胞进行修复。而在肿瘤抑制因子*Rb1*和*Trp53*缺失的情况下，小鼠体内的NE细胞能够转化成为小细胞肺癌^[12]^。

#### 1.1.2 前体细胞来源的LCSCs

与干细胞相比，前体细胞在组织中的含量明显增加，同时前体细胞也存在一定的自我更新和多向分化能力。Sasai团队^[13]^对人小气道表皮细胞进行基因编辑，联合*hTERT*的过表达、Rb和p53通路的失活、*KRAS*以及*PIK3CA*和*CYCLIN-D1*的活化，成功在裸鼠模型中转化得到了肺腺癌细胞，当增加c-Myc活性，可以得到具有干性样的低分化型肺腺癌细胞。不同于Sasai团队复杂的基因编辑实验，Wang的团队^[14]^发现镍元素可以直接诱导正常的人支气管表皮细胞发生恶性转化，同时，*SOD1*的高表达促使其中一部分细胞发生去分化获得干性样特征，这部分获得干性样特征的肿瘤细胞表现出更高的恶性程度。

#### 1.1.3 肺癌细胞通过去分化获得干性

干性细胞的分化受到c-*Myc*、*SOX2*、*Oct-4*和*TP53*等多种基因以及Wnt、Notch和Hedgehog等信号通路的调控，因此当这些与分化相关的基因或者通路活性发生改变时，细胞的分化和去分化状态之间可以相互转换^[7]^。

*EGFR*突变的肺腺癌向小细胞肺癌或者肺鳞癌的转化，是临床上肿瘤细胞获得多向分化能力从而发生组织类型转化的经典案例。研究^[15]^表明*TP53*和*RB1*的失活使肿瘤细胞获得干性样特征，具有了能够多向分化的能力，在*EGFR*活化型突变的情况下会激活更利于表皮特征的转录程序并抑制细胞向神经内分泌型转化的趋势，但当采用酪氨酸激酶抑制剂分子（tyrosine-kinase inhibitors, TKIs）抑制EGFR信号时，则大大增加了这类细胞向SCLC转化的能力。

另一个与肺癌耐药直接相关的研究热点是表皮间充质转化（epithelial-mesenchymal transition, EMT），EMT主要受到*SNAI1*、*TWIST1*和*ZEB1*等转录因子的调控，促使表皮特征的蛋白减少而间充质特征的蛋白表达增多，其调控网络与CSCs的调控之间存在很多重合。EMT既是肿瘤细胞获得干性的一个重要途径，同时也是一个重要表现^[16]^。

除此之外，包括基因突变，表观遗传修饰、代谢重编程和肿瘤微环境在内的多种内外因素都被证实可以诱导肺癌细胞发生去分化。比如，白介素-6（interleukin-6, IL-6）通过上调*DNMT1*促进了A549细胞p53和p21蛋白的甲基化，诱导出细胞的干性样功能表型；而抑制*DNMT3A*可以通过上调*CDH1*来降低Wnt/β-catenin信号活性从而抑制NSCLC的干性特征^[17, 18]^。缺氧压力下，*HOXA5*通过上调*SOX2*的转录水平诱导肺腺癌细胞发生去分化；在营养和氧气同时缺乏的情况下，间充质成纤维细胞通过旁分泌IL-6、Activin-A和G-CSF，促进肺癌细胞的去分化；CD90^+^的肿瘤相关成纤维细胞可以通过旁分泌胰岛素样生长因子Ⅱ（insulin-like growth factor Ⅱ, IGF-Ⅱ）诱导肺癌细胞的Nanog表达和干性样特征的获得；一氧化氮分子可以促进Oct-4和Cav-1复合体的形成，从而诱导肺癌细胞去分化、获得干性样特征^[19-22]^。

### 1.2 对LCSCs的鉴定

#### 1.2.1 侧群细胞

采用Hoechst 33342染色，在流式细胞仪检测下鉴定到的低染细胞群，也被称为侧群（side population, SP）细胞。SP鉴定干细胞最早是由Goodel团队在对小鼠造血干细胞研究中提出的，是基于干性样细胞高表达ABCG2和ABCB1的特征，而这两种蛋白可以将Hoechst 33342排出细胞，在染色上使得这部分细胞表现为低染^[5]^。研究者们最早就是通过SP的方法在肺癌组织和细胞系中分离并鉴定到干性样细胞亚群^[5]^。

#### 1.2.2 标志性蛋白

目前在肺癌中常常采用细胞表面标志性蛋白CD133、CD44、CD90、CD117、CD166、EpCAM（CD326）、ALDH以及EMT的标志性蛋白Nanog、SOX2和Oct-4来鉴定干性样细胞群，但是尚没有得到一致认同的标志物分子^[7, 23, 24]^。比如，Eramo^[25]^和Chen^[26]^的课题组都在肺癌组织和细胞系中证实了CD133^+^的细胞可体外成球生长，能够分化出CD133^-^的细胞，10^4^个细胞足够在免疫缺陷小鼠体内形成肿瘤，然而Qiu的团队^[27]^从H446细胞中分离到的CD133^+^与CD133^-^细胞在成球能力和分化能力上并没有显著差异，提出相比于CD133，uPAR更适合作为干性样特征标记。Masciale等^[28]^在最新的研究中对比了从肺腺癌和肺鳞癌患者组织标本中分离出的LCSCs，提出不同组织分型的NSCLCs应该采用不同的标志物。

因此，一方面研究者们常常采用多标记联合鉴定的方法，另一方面对于分离到的干性样肿瘤细胞需要进行功能验证。

#### 1.2.3 功能鉴定

对干性样肿瘤细胞的鉴定，公认的金标准是在免疫缺陷小鼠体内的成瘤能力评估，早期Al-Hajj团队通过这个模型证实了100个CD44^+^/CD24^(-/low)^的乳腺癌细胞即可形成肿瘤，与对照组之间成瘤能力差到几十倍^[29, 30]^。

体外功能验证包括成球实验和克隆形成实验。成球实验通常是将细胞置于低吸附培养皿中，以无血清培养基培养，定期添加适量生长因子，检测其形成细胞球的能力。克隆形成实验是将倍比稀释的细胞接种到普通培养皿中常规培养，一定时间之后采用甲醛固定并染色以观察克隆的数目^[28, 31, 32]^。

#### 1.2.4 干性指数评估

2018年，Malta等^[33]^来自包括哈佛、斯坦福、MD Anderson癌症研究中心等多个重要研究机构的研究者们基于癌症基因组图谱（The Cancer Genome Atlas, TCGA）中33种肿瘤的转录组数据采用机器学习的方法得到并验证了可以作为肿瘤细胞干性指数的mRNAsi。2020年Zhang的课题组^[34]^即发表了利用该指数挖掘肺腺癌干性样肿瘤细胞标志物的相关工作。通过对TCGA和GEO数据库中的数据进行整合分析，Zhang课题组的工作证实了mRNAsi的分值与肺腺癌患者的临床分期正相关，与5年生存率负相关，同时进一步鉴定到细胞周期相关基因对肺癌细胞干性样特征的重要调控作用。

## 2 肺癌干性样细胞的耐药机制

### 2.1 ABC家族转运蛋白促进了药物外排

ABCB1、ABCG2和ABCC1是多药耐药（multidrug-resistant, MDR）蛋白家族中三个最为主要的成员，广泛调控亲水物质、亲脂物质在胎盘、肠道以及血脑屏障中的转运。其中，ABCB1和ABCG2被证实在干性细胞中普遍高表达，并与多种干性样肿瘤细胞的多药耐药表型相关^[24, 35, 36]^。Chen的团队^[37]^鉴定到ABCB1高表达是NSCLC细胞HCC827、HCC4006和H1299对多西他赛（Docetaxel）耐药的主要原因，当采用依克立达（Elacridar）靶向抑制ABCB1时，能够抑制耐药细胞亚群的干性样特征，并增强对Docetaxel的敏感性。Su的团队^[38]^证实通过抑制SLC27A2，可以负向调控ABCG2的表达从而逆转肺腺癌原代细胞中干性样细胞对顺铂的耐受。

### 2.2 抗氧化能力强

CSCs中的活性氧（reactive oxygen species, ROS）水平普遍降低，主要原因之一是细胞内自由基清除系统的表达水平增高^[39]^。研究^[40]^发现，ROS与调控干细胞生物学特性的信号通路相互调控，比如，WNT配体能刺激NADPH氧化酶（NADPH oxidase, NOX）家族成员NOX1产生ROS，产生的ROS又促进了核氧化还原蛋白（nucleoredoxin, NRX）的氧化，破坏了NRX与蓬乱（Dishevelled, DVL）蛋白的相互作用，与NRX解离的DVL能增加β-catenin的稳定性，继而增加其与T细胞因子（T cell factor, TCF）家族转录因子的相互作用，进一步调控WNT的靶基因，或者与叉形头转录因子O亚型（forkhead box protein O, FOXO）家族作用，调控细胞的还原状态。Lei的团队^[41]^证实，改变细胞内的ROS水平可以通过激活Nrf2诱导Notch信号的活化，从而促进基底干细胞的自我更新，与此同时激活细胞内的抗氧化途径来减少细胞内的ROS，以保持细胞内的氧化还原稳态。ALDH1可以通过上调SOD2和GPX4来调控ROS-RCS代谢通路，以平衡厄洛替尼引起的细胞内ROS毒性。

由ROS水平升高诱导细胞凋亡是多种药物毒性的重要途径之一，CSCs中低水平的ROS，以及增强的氧化还原平衡能力，加强了其对治疗的耐受性。这种还原调控机制对于干细胞的生物学特征、肺损伤修复以及肿瘤等疾病具有重要的研究价值^[42]^。

### 2.3 凋亡逃逸

CSCs可以通过多种机制来降低药物引起的细胞凋亡，包括改变Bcl-2家族中抗凋亡蛋白和促凋亡蛋白之间的平衡，抑制线粒体介导的凋亡通路，促进凋亡抑制因子的表达等^[24, 30, 43]^。Zeuner和同事^[44]^证实了从肺癌组织中提取出的LCSCs普遍高表达Bcl-X_L_，当采用抑制剂分子ABT737时能够有效诱导干性样细胞的凋亡、阻断LCSCs移植瘤的生长。Zakaria的团队^[45]^也报道了NSCLC细胞系中的CSCs高表达抗凋亡蛋白BCL-2、Bcl-X_L_和凋亡抑制因子survivin，并且核因子κB（nuclear factor kappa-B, NF-κB）信号在其中起到了关键调控作用。

此外，与正常成体干性细胞一样，干性样肿瘤细胞也会通过进入休眠状态来维持组织平衡，并且在微环境不利于其生存的情况下，起到自我保护的作用^[46]^。休眠状态的细胞周期一般停滞在G_0_期，这就降低了对靶向细胞快速增殖特征的传统化疗药物的敏感性，同时，休眠的LCSCs被证实促凋亡蛋白活性显著降低，并能通过自噬和内质网应激等途径适应性生存^[24, 46, 47]^。

### 2.4 DNA损伤修复能力增强

研究发现，CSCs的DNA损伤修复能力很强。DNA损伤的主要感受蛋白包括ATM和ATR相关的蛋白激酶，ATM识别双链DNA断裂，ATR识别单链断裂。当DNA发生损伤，ATM和ATR激酶会与PARP-1、BRCA1形成复合体，磷酸化CHK1和CHK2，继而促进下游靶蛋白的活化，包括p53和CDC25A，促进细胞周期停滞，给细胞足够的时间进行DNA损伤修复或者诱导凋亡。目前认为与CSCs的耐药有关的DNA修复蛋白主要包括CHK1、CHK2、ATR、MSI1、RAD50和RAD51。

Bartucci团队^[48]^验证到，由NSCLC患者组织就分离到的干性样肿瘤细胞，在化疗药物引起的损伤应答过程中，CHK1是最早也是最显著的活化指标，并且不依赖于p53的状态。而在已经分化的肺癌细胞中，CHK1的活化很微弱。体内、体外采用CHK1的抑制剂联合化疗能够显著地抑制干性样肺癌细胞。

Yu的团队^[49]^对比了干性样和非干性样肺腺癌细胞在顺铂作用下的DNA损伤修复能力，证实前者细胞中DNA损伤更小，可能是由于AQP2和CTR1的下调，因此具有对顺铂引起的DNA双链间的损伤修复能力更强。

### 2.5 与肿瘤微环境的相互影响

多项研究证实，CSCs与其所处的微环境之间存在相互调控关系。如前所述，营养和氧气的缺失、炎性反应，以及药物作用等都可能促进肿瘤细胞通过去分化获得干性。此外，细胞外基质的改变可以通过应力传导影响细胞骨架、形态和粘附力，并调控在CSCs干性维系中发挥重要作用的转录因子YAP/TAZ的活性。YAP/TAZ的靶基因中包括了参与EMT调控的重要蛋白，而同时细胞EMT过程中伴随的形态与粘附力等的改变又作为上游调控因子直接影响了YAP/TAZ的活性^[50]^。近来不少研究者们提出，YAP/TAZ的磷酸化能够对非干性肿瘤细胞重编程，使其具备完整的CSCs属性^[50, 51]^。Kurppa等^[47]^通过试验证实LCSCs在药物的作用下可以通过启动YAP介导的重编程进入休眠状态而逃避凋亡，Pisanu的团队^[52]^也提到在LCSCs中硬脂酰辅酶A脱氢酶SCD1主要是通过调控YAP/TAZ的活性维系细胞的干性特征以及对顺铂的耐受表型。

此外，CSCs的自我更新和多向分化能力会造成肿瘤细胞的异质性，并且抑制肿瘤微环境对免疫细胞的招募^[53, 54]^。

## 3 靶向肺癌干性样细胞逆转耐药的策略

### 3.1 诱导分化

Zhai的团队^[55]^证实神经表皮生长因子样蛋白NELL1能够通过抑制c-MET-Notch信号诱导LCSCs的分化，Huang的团队^[56]^证实绿原酸（chlorogenic acid）可以增强*SUMO1*的表达，引起c-Myc的类泛素化继而促进LCSCs的分化。这些研究结果表明，通过诱导LCSCs的分化可以有效降低肿瘤细胞的成瘤能力、增强肿瘤细胞对药物的敏感性。

### 3.2 靶向调控细胞干性的关键蛋白和信号通路

早期用来戒酒的双硫仑（Disulfiram）能够抑制ALDH活性，近来在包括肺癌在内的多种肿瘤中被证实具有抑制肿瘤的作用^[57]^。Ouyang等^[58]^在实验中鉴定到芳香烃受体（aryl hydrocarbon receptor, AhR）可以通过与干性特征基因ABCG2、KLF4和c-Myc启动子的结合来促进这些基因的表达，而小分子抑制剂TCID可以通过靶向抑制泛素羧基末端水解酶L3的活性，增强AhR的降解，最终达到抑制LCSCs的效果。

一些靶向Wnt、Notch、Hedgehog和Hippo信号通路的单克隆抗体分子已经进入了临床试验阶段，包括DLL4的抗体Demcizumab、Wnt/Fzd的广谱抗体Vantictumab、Hedgehog通路的抗体Taladegib和Saridegib，以及靶向Hippo通路的Pevonedistat等，但是效果和毒性仍然有待评估。由于这些调控CSCs干性的蛋白和信号通路同样也在正常成体干细胞中发挥重要功能，因此在临床应用中需要更加谨慎^[4]^。

值得关注的是，姜黄素和异硫氰酸盐等天然药物成分，在靶向LCSCs中同样表现出了重要潜力。比如，姜黄素能有效抑制LCSCs的Wnt和Hedgehog通路，而异硫氰酸盐能够通过调控miR-214，继而抑制MYC的转录^[59, 60]^。

### 3.3 靶向肿瘤微环境

钙调蛋白的抑制剂分子CBP501，在小鼠模型中可以抑制巨噬细胞分泌的IL-6、IL-10和肿瘤坏死因子（tumor necrosis factor, TNF），从而阻断了肺腺癌中ABCG2的表达及细胞的成球能力和成瘤能力，限制了肺癌细胞的转移^[61]^。阿帕替尼近来在晚期肺癌患者的Ⅱ期临床试验中表现良好，研究者们发现阿帕替尼可以通过靶向血管内皮细胞生长因子受体2（vascular endothelial growth factor receptor 2, VEGFR2）降低肿瘤微环境中巨噬细胞诱导的肺癌细胞EMT重编程而达到有效抑制肺癌发展的效果^[62]^。

## 4 小结与展望

LCSCs是一群具有自我更新、多向分化和无限增殖能力的细胞亚群，被提出并证实是肺癌发生、耐药、复发和转移的主要原因。LCSCs既能由正常成体干细胞或前体细胞恶性转化而来，也可由普通肺癌细胞发生去分化获得干性而产生。具有干性特征的肺癌细胞由于其较强的药物外排与解毒能力、增强的抗凋亡与DNA损伤修复能力，以及对肿瘤微环境的适应与调节能力，对传统化疗、靶向治疗和免疫治疗均表现出了耐受，同时，在肿瘤的异质性调控中也发挥了重要作用。因此，鉴定和靶向LCSCs是临床克服耐药与复发的关键之一。

LCSCs的干性主要受到Wnt、Notch、Hedgehog和Hippo通路以及*SOX2*、*c-Myc*、*Oct-4*和*TP53*等基因的调控，并受到氧化还原水平和肿瘤微环境中细胞外基质（extracellular matrix, ECM）与多种细胞因子的影响，然而由于LCSCs与正常成体干细胞和前体细胞具有相似的调控网络，鉴定到其特异性调控靶点对于临床具有重要的意义。同时，由于LCSCs对药物分子的高外排能力，CSCs的特异性药物运载方法对于提高药物吸收也很有帮助。

LCSCs的调控因素复杂，并受到微环境的多重影响，因此在研究过程中需要更加接近人体肿瘤实际结构与微环境的培养模式，包括肿瘤细胞的立体生长、多种细胞的共培养等，人源肿瘤异种移植（patient-derived xenografts, PDX）模型、类器官以及更新的仿生系统能够更好地增加研究结果的可靠性。同时，干性样肿瘤细胞的异质性、细胞分化状态的可逆性和调控机制的多样性，增加了肺癌临床对精准医疗的需求，以及依靠无创技术对患者进行长期监测的需求。
